# The Role of Glutamate Receptors in Epilepsy

**DOI:** 10.3390/biomedicines11030783

**Published:** 2023-03-04

**Authors:** Tsang-Shan Chen, Tzu-Hsin Huang, Ming-Chi Lai, Chin-Wei Huang

**Affiliations:** 1 Department of Neurology, Tainan Sin-Lau Hospital, Tainan 70142, Taiwan; 2 Zhengxin Neurology & Rehabilitation Center, Tainan 70459, Taiwan; 3 Department of Pediatrics, Chi-Mei Medical Center, Tainan 71004, Taiwan; 4 Department of Neurology, National Cheng Kung University Hospital, College of Medicine, National Cheng Kung University, Tainan 70142, Taiwan

**Keywords:** glutamate, AMPA, NMDA, kainite, receptor, metabotropic, epilepsy

## Abstract

Glutamate is an essential excitatory neurotransmitter in the central nervous system, playing an indispensable role in neuronal development and memory formation. The dysregulation of glutamate receptors and the glutamatergic system is involved in numerous neurological and psychiatric disorders, especially epilepsy. There are two main classes of glutamate receptor, namely ionotropic and metabotropic (mGluRs) receptors. The former stimulate fast excitatory neurotransmission, are *N*-methyl-d-aspartate (NMDA), α-amino-3-hydroxy-5-methyl-4-isoxazole propionic acid (AMPA), and kainate; while the latter are G-protein-coupled receptors that mediate glutamatergic activity via intracellular messenger systems. Glutamate, glutamate receptors, and regulation of astrocytes are significantly involved in the pathogenesis of acute seizure and chronic epilepsy. Some glutamate receptor antagonists have been shown to be effective for the treatment of epilepsy, and research and clinical trials are ongoing.

## 1. Introduction

Epilepsy is a brain disease associated with chronic recurrent seizures. The mainstay theory is that it is caused by an imbalance between the excitatory and inhibitory conductance of nerve cells within the brain [1]. Glutamate is the major neurotransmitter for excitatory neuronal signaling, while γ-aminobutyric acid (GABA) has an inhibitory function. Glutamate plays a role in both pre- and post-synaptic excitatory neurotransmission resulting in cellular and network hyperactivity, and underlies the formation of ictogenesis and epileptogenesis. Exploring and understanding the glutamatergic mechanisms will contribute to the improvement of epilepsy management strategies. This article reviews the current literature on the functions of glutamate receptors and the modulation of glutamate transmission.

## 2. Subcellular Structure and Physiology of Glutamate Receptors

Glutamate mediates excitatory transmission via various pre- and post-synaptic glutamate receptors. The glutamate receptors encompass ionotropic receptors (transmembrane ligand-gated ion channels) and metabotropic receptors, which affect presynaptic glutamate release or indirectly modulate the function of ionotropic receptors. Ionotropic glutamate receptors (iGluRs) share a common tetramer containing A–D subunits [2,3]. Presynaptic neuronal depolarization drives calcium-dependent glutamate release from terminal vesicles to the cleft of the synapse. iGluR ion channels open in response to glutamate binding, allowing sodium with calcium cations to flow in, which rapidly depolarizes the postsynaptic membrane and initiates signal transduction in the postsynaptic neuron [4]. As high levels of extracellular glutamate have an excitotoxic effect in neurons, the glutamate concentration is optimally controlled via the reuptake of glutamate from the synapse by astrocytic glutamate transporters, including glutamate–aspartate transporter (GLAST) and glutamate transporter-1 (GLT-1) [5,6,7]. When glutamate is taken up by astrocytes, it is converted to glutamine by the enzyme glutamine synthetase. The glutamine is then delivered from the astrocytes to neurons via glutamine transporters. Finally, glutamine is converted to glutamate in the neurons by glutaminase to complete the glutamate–glutamine cycle [8]. Glutamate is also involved in long-term potentiation and synaptic plasticity in the normal brain [9]. Three kinds of postsynaptic ionotropic glutamate receptors have been identified, namely the α-amino-3-hydroxy-5-methyl-4-isoxazolepro-pionic acid (AMPA) receptors (AMPARs), which are responsible for fast excitatory neurotransmission, *N*-methyl-D-aspartate (NMDA) receptors (NMDARs), which mediate most of the slow postsynaptic excitatory potentials, and the kainate receptor (KAR). The function of KAR is not clearly known, although it may modulate pre- and post-synaptic excitatory neurotransmission [10]. Injection of kainate into the rat hippocampus mediated the inhibition of presynaptic GABA release and postsynaptic KAR activation of glutamatergic neurons, resulting in epileptogenesis [11,12].

### 2.1. AMPA Receptors (AMPARs)

**Figure 1:**
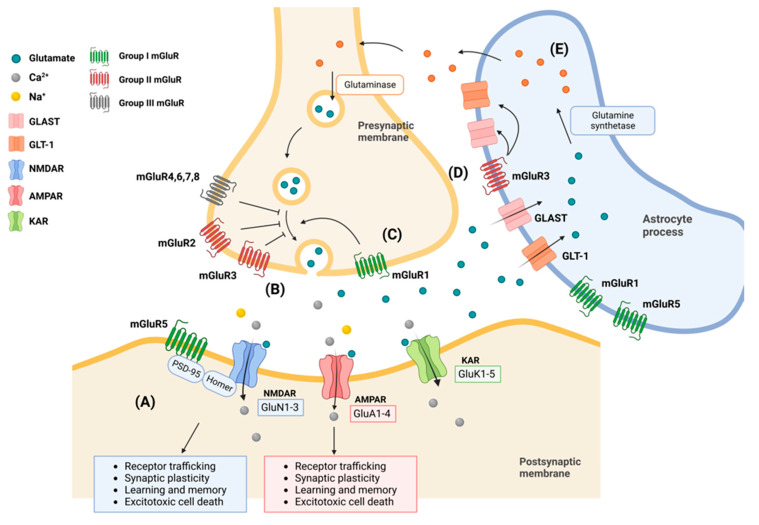
Mechanism and types of ionotropic and metabotropic glutamate receptors with associated proteins. (**A**) Ionotropic glutamate receptors are composed of four subunits and are structured similarly to a central ion channel pore, and are activated by NMDAR (GluN1–3 subunits), AMPAR (GluA1–4 subunits), and KAR (GluK1–5 subunits). These channels regulate Ca^2+^ permeability and play roles in receptor trafficking, synaptic plasticity, learning, memory, and even cell death. (**B**) Metabotropic glutamate receptors are G-protein-coupled receptors that modulate synaptic transmission and neuronal excitability. They are divided into three groups: Group I includes mGluR1 and 5; Group II includes mGluR2 and 3; Group III includes mGluR4, 6, 7, and 8. (**C**) Group I metabotropic receptors enhance the excitatory function in neurons. Presynaptic mGluR1 facilitates vesicular release of glutamate, and postsynaptic mGluR5 modulates the action of postsynaptic NMDARs via PSD-95 and Homer protein, leading to phosphorylation of NMDARs and potentiation of NMDAR currents. (**D**) Group II and Group III metabotropic receptors mainly inhibit excitatory function. mGluR2 and 3 and mGluR4, 6, 7, and 8 inhibit presynaptic glutamate release. mGluR3 is also expressed on astrocytes, positively modulating the glutamate transporter proteins GLAST and GLT-1, enhancing synaptic glutamate reuptake, and thus countering the hyperexcitability of neurons. (**E**) Glutamate in the synaptic cleft is taken up by astrocytes around the synapses, and is transformed to glutamine by glutamine synthetase; then, glutamine is transformed to glutamate and stored in vesicles of the presynaptic terminals to maintain neuronal communication (Figure created with BioRender.com, accessed on 23 February 2023).

Glutamate is the direct binding ligand that activates AMPARs. AMPARs are expressed in the postsynaptic neuronal membrane and are involved in rapid excitatory neurotransmission in the brain. They have a tetrameric configuration containing four types of subunit, GluA1 to GluA4 (also known as GluR-A to GluR-D), assembled in different combinations [13] (Figure 1). The GluA2 subunit is the key site for regulating calcium permeability [14,15]. The calcium permeability regulation of the GluA2 subunit is affected by a posttranscriptional modification. The AMPARs are calcium (Ca^2+^)-permeable if they contain the unedited GluA2 subunit or the GluA2-lacking structure [16,17]. Most AMPARs in the brain contain the edited GluA2 subunit and are therefore Ca^2+^-impermeable. Mounting evidence suggests that Ca^2+^-permeable AMPARs play an important role in receptor trafficking, synaptic plasticity, learning, and memory [18,19,20,21,22], as well as excitotoxic cell death [23,24]. In other words, the synaptic homeostatic plasticity comes from dynamic changes to AMPARs that occur during their biosynthesis, membrane trafficking, and degradation, which are controlled by complex regulating proteins [20]. In experimental rats following pilocarpine-induced status epilepticus (SE), the expression of calcium-impermeable GluA2-containing AMPARs in their cortex was increased, indicating a potential long-term change in neuroplasticity for neuroprotection in response to SE-induced neurotoxicity [25].

### 2.2. N-Methyl-D-Aspartate Receptors (NMDARs)

As glutamate release activates glutamate receptors, it mediates an inward cation current and thereby depolarizes the postsynaptic neurons. Two types of excitatory postsynaptic currents can be found. AMPARs activation mediates a fast current with a rapid increase and fades away, whereas the NMDARs mediates a slower current that lasts from tens to hundreds of milliseconds [26,27,28]. NMDARs also have a tetrameric structure made up of different combinations of GluN1, GluN2, and GluN3 subunits, with each having several isoforms [29] (Figure 1). The GluN1 subunit is essential for all functional NMDARs and has binding sites for glycine and D-serine. Glycine (B)-binding sites in both GluN1 and GluN3A subunits facilitate receptor forward trafficking to the cell surface. Mutation in the glycine-binding site of the human GluN3A subunit remarkably reduces the surface expression of NMDARs [30]. These findings indicate that glycine-binding sites play an important role in the regulation of NMDARs. The GluN2 subunits provide glutamate-binding sites and control the expression and functional properties of NMDARs [31]. In brief, several types of NMDAR modulators are noted. They include competitive antagonists acting at the agonist binding site, sodium and calcium blockers, allosteric sites different from endogenous agonist binding sites, and glycine site antagonists [32]. The binding of glutamatergic ligands is not sufficient to open the channel because the NMDAR channel is plugged by magnesium ion (Mg^2+^) at the resting membrane potential. When the Mg^2+^ is removed, the membrane is depolarized, allowing a voltage-dependent inward flow of sodium (Na^+^) and Ca^2+^ ions while potassium (K^+^) ions flow out of the cell [33]. Low extracellular magnesium is associated with seizure-like discharges in the brain due to the unblocking of NMDARs [34]. Calcium influx through NMDARs can activate the binding proteins and is thought to play an important role in synaptic plasticity, a cellular mechanism for learning and memory [35,36,37].

### 2.3. Kainate Receptors

Kainate receptors (KARs) are ionotropic glutamate receptors, in addition to AMPA and NMDARs. KARs are activated by the agonist, kainate, and play a role in postsynaptic excitatory neurotransmission. KARs are also tetrameric structures by different assemblies of Glu K1-Glu K5 to form functional KARs. Glu K1, K2, and K3 may undergo editing and splicing to form some variants such as GluK1a, GluK1b, GluK1c, and GluK2a-2c etcetera [38]. KARs exert their intracerebral expression in the amygdala, entorhinal cortex, and hippocampus [39,40,41]. They generate a stereotypical electrophysiological response when activated, which consists of a small amplitude and slow decay current, resulting from both their interaction with their auxiliary subunits and the biophysical properties of heteromeric receptors [42]. KARs mediate both canonical ionotropic and non-canonical metabotropic signaling. The KAR activation of a non-canonical signaling pathway is analogous to metabotropic glutamate receptors, which also modulate neuronal excitability and transmitter release. Among the multiple mechanisms in the regulation of neurotransmitter release by presynaptic KARs, some are considered to involve a non-canonical action of KARs [43]. KARs could act through a non-canonical mode of action through activation of second messenger signaling pathways [43,44].

In temporal lobe epilepsy, aberrant recruitment of KARs at recurrent mossy fiber synapses participates in epileptogenic neuronal activity [44]. In addition, kainic acid (KA) can be used to establish an experimental model of temporal lobe epilepsy (TLE), which is a common type of partial epilepsy characterized by neuronal loss in the CA1 and CA3 regions of the hippocampus. Intra-amygdaloid injections of KA induce psychomotor seizures and produce neuropathological lesions similar to those found in TLE [45,46]. Furthermore, intra-hippocampal injection of KA also produced similar preconditions for TLE, such as old cerebral insults, encephalitis, or SE [47]. KA1 subunits are highly expressed in CA3 pyramidal cells and KA2 subunits are highly expressed in both CA1 and CA3 pyramidal cells [48,49,50]. Therefore, KA1 and KA2 receptors have high affinity for glutamate and high expression in the CA3 region of the hippocampus. This is why this region is vulnerable to the excitotoxic damage induced by KA, and the hippocampus often becomes the epileptogenic zone in this activation model [51,52].

Although the mechanism of KAR is least known in the iGluRs, there is some progress of advanced knowledge in recent years. As mentioned earlier, KARs act by modulating neuronal excitability and regulation of synaptic network activity by complex expression patterns in the CNS [53]. The retention of KARs in the endoplasmic reticulum (ER) or trafficking of KARs from the ER to the cell surface is an important regulation mechanism. Some KAR-interacting proteins are involved in the surface trafficking. For example, NETO (neuropilin and tolloid-like) proteins possibly play a role in the trafficking of KARs. Other protein reacting substances such as PKC (protein kinase C)-mediated phosphorylation and SUMOylation (small ubiquitin-related modifier) have been reported to have impacts on surface expression and endocytosis [54]. Moreover, molecular modeling and calcium imaging studies showed that the occupancy of both GluK2 and GluK5 ligand binding domains is required for the full activation of GluK2/GluK5 heteromeric KAR channels [55]. Of note, the non-canonical signaling of KARs which activates phospholipase C and PKC via a metabotropic G-protein dependent pathway, regulating neuronal excitability by inhibiting the slow afterhyperpolarization, neurotransmitter release, and glutamate receptor trafficking could potentially contribute to increased neuronal excitability [56]. Both of the roles of the canonical and non-canonical KARs pathways in the generation of seizure activity are worth further investigation.

The biosynthesis, assembly, and cell surface trafficking of KARs are key determinants of neuronal excitability in the CNS. As KARs dysfunction is linked to ischemic, chronic pain, epilepsy, and psychiatric disease such as schizophrenia [10], knowing the action of KAR-interacting proteins in the trafficking and surface expression to develop new therapeutic strategies against these diseases is important.

### 2.4. Metabotropic Receptors

Metabotropic receptors (mGluRs) are G-protein-coupled non-ionotropic receptors that modulate either presynaptic glutamate release or postsynaptic ionotropic receptors. There are three major functional subgroups of mGluRs: Group I mGluRs encompass presynaptic mGluR1, postsynaptic mGluR5, and both in astrocytes. Group I mGluRs enhance excitatory function in mGluR1 and mGluR5. Presynaptic mGluR1 facilitates the vesicular release of glutamate, whereas postsynaptic mGluR5 can modulate the action of postsynaptic ionotropic receptors.

Activation of mGluR1 by a specific agonist can maintain and prolong interictal discharge [57]. mGluR1 can also be activated by increases in intracellular calcium and depolarization of hippocampal CA1 pyramidal cells, which are involved in inducing long-term potentiation (LTP) and long-term depression (LTD), a long-lasting synaptic plasticity, at multiple glutamatergic synapses [58]. Upregulation of hippocampal mGlu5 was found in brain slices from pharmaco-resistant TLE patients. The upregulation of mGluR5 in surviving neurons is probably a consequence of the hyperexcitability of the hippocampus, but not the cause of epileptic seizures in pharmaco-resistant TLE patients, as this phenomenon has been observed in both hippocampal sclerosis and non-hippocampal sclerosis patients [59]. Additionally, protein–protein interaction is an important coupling mechanism for the action and regulation of metabotropic receptors. mGlu5 binds to NMDARs via scaffolding proteins, both postsynaptic density protein 95 (PSD-95) and Homer protein, leading to phosphorylation of NMDARs. The net result is potentiation of NMDAR currents [58,60]. Group II metabotropic receptors include mGluR2/3, which are predominately located on the presynaptic terminal and can inhibit presynaptic glutamate release. mGluR2/3 expression was markedly and progressively down-regulated in both CA1 and CA3 in a pilocarpine-induced SE model [61,62]. mGluR3 is expressed on astrocytes and positively modulates GLAST and GLT-1, the glutamate transporter proteins for synaptic glutamate reuptake, and consequently counters hyperexcitability [63]. Interestingly, astrocytic mGluR3 and mGluR5 expression are upregulated in TLE, indicating that seizure-induced upregulation of two opposite mGluR subtypes in reactive astrocytes is a novel mechanism for modulation of glial function and changes in glial–neuronal communication in the course of epileptogenesis [64]. Group III metabotropic glutamate receptors include mGluR4, 6, 7, and 8, and function as inhibitory presynaptic receptors. In an animal model of TLE, mGluR1 can promote seizure susceptibility, whereas mGluR4 is upregulated to counteract the excitatory activity and seizure-associated vulnerability of hippocampal neurons [65]. In summary, metabotropic glutamate receptors modulate glutamatergic signaling by altering their expression and distribution, and play key roles in hippocampal excitability, epileptogenesis, and neuronal degeneration.

### 2.5. Astrocytes in Glutamate Uptake and Release

Astrocytes act on the uptake of synaptic and extracellular glutamate by astrocytic glutamate transporters. Several studies over the past 10 years show that glutamate is released from astrocytes for regulation of neuronal activity under physiological conditions [66]. When glutamate is released from excitatory neurons, it stimulates group I metabotropic glutamate receptors in astrocytes. The activation of these receptors induces elevated release of the astrocytic intracellular Ca^2+^ concentration, which in turn triggers glutamate release from astrocytes. The astroglial-released glutamate subsequently activates the extrasynaptic GLuN2B subunit of NMDARs in the nearby neurons, generating slow inward currents inside these neurons that synchronize their action potential firing [67,68,69,70]. The astroglial-released glutamate also stimulates presynaptic group I mGluRs and NMDARs, inducing presynaptic neurons to release more glutamate [71,72,73]. This glial–neuronal crosstalk can possibly explain why astrocytes are capable of generating seizures by impairment of homeostatic extracellular glutamate [74]. On the other hand, glutamate also potentiates neuronal inhibition. Inhibitory interneurons release GABA, which activates astrocytic GABA_B_ receptors and induces an increased release of Ca^2+^ within astrocytes, which in turn triggers glutamate releases [75]. Glutamate acts on presynaptic ionotropic glutamate receptors and augments the release of more GABA from the surrounding inhibitory neurons [76]. Therefore, astrocytes act through a unique dual function involving both glutamate uptake and release to maintain glutamate homeostasis in the CNS.

### 2.6. Cannabidiol (CBD) and Glutamate Signaling

Cannabidiol (CBD) is a compound extracted from cannabis, which has been proven to have multiple pharmacological effects, including anti-inflammatory [77,78,79], immunomodulatory [80], neuroprotective [81,82], and anti-seizure effects [83,84]. It could induce neuroprotection through the normalization of homeostasis of glutamate [85]. Evidence shows that CBD modulates glutamate and GABA levels in brain regions such as the basal ganglia and the dorsomedial prefrontal cortex [86]. In the presynaptic nerve terminal, a transmembrane orphan G protein-coupled receptor 55 (GPR 55) can be activated by agonists to increase intracellular Ca^2+^ concentration and facilitate glutamate release from vesicles [83]. GRP 55 is one of the targets on which CBD acts. CBD has been shown to antagonize GRP 55, thereby decreasing glutamine release and exerting its anti-seizure effect [84,87]. Furthermore, the overexpression of cannabinoid type 1 (CB1) receptors in hilar mossy cells of the dentate gyrus and in pyramidal cells of the hippocampal regions was shown to significantly reduce the severity of kainate-induced SE [88] and the expression of CB1 receptors in excitatory neurons of the cerebral cortex, hippocampus, and amygdala of the global CB1 receptor knockout mice was able to prevent the exacerbation of kainate-induced seizures [89,90]. Clinically, CBD has been demonstrated to reduce seizure frequency in drug-resistant epilepsies, including Dravet syndrome, Lennox-Gastaut syndrome, and tuberous sclerosis complex [91,92,93,94,95]. In conclusion, glutamate plays a crucial role in the majority of excitatory transmission in the CNS via ionotropic and metabotropic glutamate receptors. The three ionotropic glutamate receptors (NMDAR, AMPAR, KAR) share similar tetramer structures, with slow excitatory transmission associated with NMDAR and fast excitatory transmission with AMPAR. The inward cation current regulation, including Ca^2+^ and Na^+^, may contribute to synaptic plasticity, learning, and memory in the normal brain. Metabotropic glutamate receptors are G-protein-coupled receptors, containing three subgroups that mediate glutamate release in the presynapse, ionotropic receptors in the postsynapse, and glutamate recycling in astrocytes. Alterations in their expression and distribution may play a role in the development of epilepsy or neurodegeneration.

## 3. Main Mechanism of Glutamate Receptor in Epilepsy

Activation of the NMDAR has been demonstrated to evoke burst firing in CA1 neurons and granular cells of the hippocampus [96,97]. It has shown that both NMDA and quisqualate, an AMPA agonist, evoke a distinct spike in activity. Quisqualate induces a rapid depolarization that evokes tonic firing, whereas NMDA produces bursts of fast action potentials superimposed on an underlying depolarizing shift of membrane potential [98]. From animal models of epilepsy and SE in humans, it is evident that glutamate constitutes a key role in seizures and epileptogenesis. SE-induced glutamate release results in over-stimulation of glutamate receptors, sustained prolonged seizure activity, and seizure-related excitotoxic brain injury [99,100].

Region-specific alterations of glutamate receptor subunits in a pilocarpine model of TLE suggest cellular mechanisms contributing to a generation of independent epileptogenic networks in different temporal lobe areas [101]. The role of NMDAR-mediated excitotoxicity, primarily via excessive Ca^2+^ influx through the extrasynaptic GluN2B-containing receptors, in the context of both acute and chronic neurological disorders, is well-established [56,102,103,104,105,106], and the carboxy terminal domain of GluN2B remains an important factor mediating excitotoxic signaling [103]. In addition, interfering peptides disrupting the coupling of GluN2B to PSD-95 and nNOS [107,108], reducing nNOS activation and nitrosative and oxidative stresses, entered clinical trials and showed effectiveness in terms of clinical outcome [109,110]. The diversity of the carboxy terminal domain among different GluN2 subunits is therefore important in determining the subunit-specific trafficking of GluN2-containing NMDARs and excitotoxicity in various neurological disorders.

A well-established mechanism of regulation of receptor surface expression is the phosphorylation/dephosphorylation of specific residues in the carboxy terminal domain of GluN2A and GluN2B subunits. As we know, a key mediator of neuronal excitotoxicity is the excessive activation of NMDARs and the calcium influx. It has been demonstrated that the activation of synaptic versus extrasynaptic NMDAR stimulation is remarkably different in the context of excitotoxicity, possibly related to the different subunit composition of the two receptors [111]. Importantly, studies also provided strong evidence that the carboxy terminal domain of GluN2 subunits and their C-terminal interacting proteins play roles in controlling the subcellular localization, synaptic targeting, and intracellular Ca^2+^ signaling of GluN2-containing NMDARs, and affect neuronal excitotoxicity [56,111].

With prolonged SE, receptor trafficking is noted with GABA receptors being internalized and NMDARs migrating to neuronal synapses [112,113]. Overactivation of AMPARs can elicit TLE. There is typically relatively dense expression of AMPARs in the hippocampus in this type of chronic seizure [114,115]. These findings show that glutamate receptor expression and distribution are dynamic. These SE-induced changes in receptor localization explain why drugs that target GABAergic neurotransmission (such as benzodiazepines) usually fail to control refractory SE, whereas NMDAR antagonists in combination with GABA agonists can often alleviate sustained SE. Sodium and calcium channels also exert indirect effects in regulating glutamate concentration and neuron protection. Presynaptic calcium and sodium channel activation enhance glutamate efflux into the extracellular space, and a higher concentration of glutamate subsequently leads to neuronal injury [116]. Riluzole, a neuroprotector, can lessen the symptoms of motor neuron disease by blocking persistent calcium and sodium currents, thereby modulating glutamate release via presynaptic NMDARs [117].

One of the main mechanisms for the induction of seizures is the suppression of GABA release when KA activates KAR, along with postsynaptic KAR activation of glutamatergic neurons [118,119]. Interneurons in the CA1 region of the hippocampus have KARs containing the GluK1 subunit in their axonal compartment and GluK2 in the somatodendritic compartment [120]. KARs containing the GluK2 subunit have been linked to limbic epilepsy, related to their specific expression in CA3 pyramidal neurons [49]. In animal models of TLE and in human patients, neuronal networks undergo a substantial reorganization whereby neuronal death, sprouting, and aberrant connection are prominent [121]. GluK1 mRNA is abundant in temporal lobe areas, including the amygdala, and prolonged activation of basolateral GluK1 subunit-containing KARs by ATPA (an agonist of GluK1 subunit-containing KARs) induces spontaneous epileptiform activity, which is sensitive to KAR antagonism [119,122].

NMDAR antagonists such as MK-801 or ketamine have antiseizure effects and provide neuroprotection against excitotoxicity after prolonged SE [123,124,125]. After prolonged SE, NMDARs mediate long-term remodeling of synaptic connectivity and dendritic morphology, thereby increasing excitatory signaling and promoting the onset of spontaneous recurrent seizures. Moreover, NMDAR subunits undergo posttranscriptional modifications and trafficking of GluN1 subunits from the intracellular compartment to the cell membrane surface in response to SE [113,126]. Brain-specific microRNA-134, a small non-coding RNA, is localized to the synaptodendritic compartment of rat hippocampal neurons and participates in NMDAR-dependent spine remodeling, which reinforces postsynaptic sites of excitatory transmission [127]. Inhibition of microRNA-134 is a promising mechanism for the development of antiseizure medications [128].

### 3.1. NMDA Receptors Mutation

**Figure 2:**
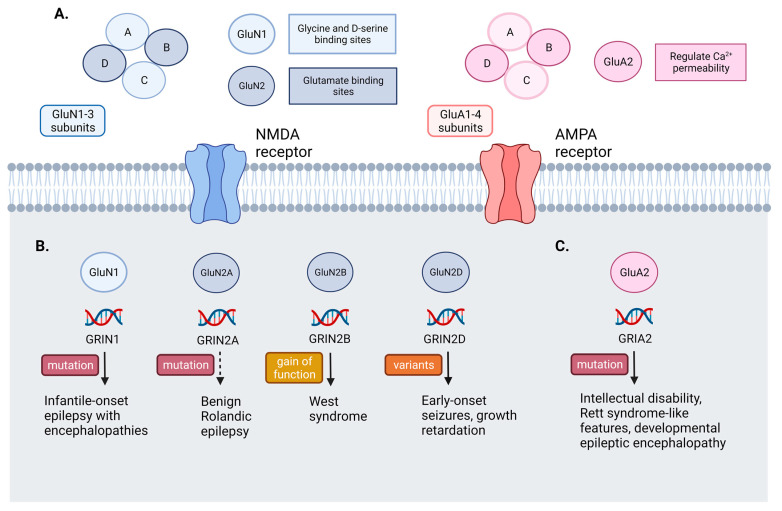
Structures and possible mutations of ionotropic glutamate receptors with associated clinical features. (**A**) Ionotropic glutamate receptors such as NMDA and AMPA receptors share a common tetramer containing A–D subunits in different combinations of subunits. In NMDA receptors, the GluN1 subunit is essential for all functional NMDARs and has binding sites for glycine and D-serine. The GluN2 subunits provide glutamate-binding sites and control the expression and functional properties of NMDARs. In AMPA receptors, the GluA2 subunit is the key site for regulating calcium permeability. (**B**) Mutations in the subunits have been found to cause clinical syndromes. GRIN1 missense mutations can cause infantile-onset epilepsy with encephalopathies. GluN2A mutations are related to benign Rolandic epilepsy. GRIN2B gain-of-function mutation causes West syndrome. Some GRIN2D variants were reported with initial early-onset seizures. (**C**) The heterozygous mutations of GRIA2 gene encoding GluA2 subunit were found to have phenotypes of intellectual disability, neurodevelopmental abnormalities, and seizure (Figure created with BioRender.com, accessed on 23 February 2023).

Some gene mutations have been identified in NMDARs. Mutations in GRIN1 (encodes the GluN1 subunit), GRIN2B (encodes the GluN2B), and GRIN2D (Glu N2D) present with severe clinical phenotypes, including severe epilepsy with mental retardation and developmental delay (Figure 2). Four de novo missense GRIN1 mutations cause infantile-onset epilepsy with encephalopathies, as well as hyperkinetic movement disorders [129]. GRIN2B gain-of-function mutation causes West syndrome, presenting with focal epilepsy and intellectual disability [130]. Six new GRIN2D variants were reported with initial early-onset seizures (by one year old) and general growth retardation, and some had low intraocular pressure, dyskinesia, or autistic behavior [131]. GluN2A mutations or variants can be related to benign Rolandic epilepsy [132,133]. Notably, the excitatory glutamatergic neurons and synapses outweigh the amount of GABAergic neurons and synapses [134,135]. A similar epilepsy phenotype may exist in a state of hyper-NMDA function (excessive NMDAR activation, or NMDA-pathies) or hypo-NMDA function, which diminishes GABA interneuron activation and thereby causes disinhibition [136]. For this reason, either increased or decreased NMDA function will enhance neuronal excitation. Hypofunction of NMDARs is also found in the aging brain and persistent hypofunction will cause neurodegeneration with cognitive and psychotic impairment, in addition to epilepsy [137].

### 3.2. AMPA Receptors Mutation

Mutation of AMPARs is less common than NMDARs. AMPAR gene mutations are often associated with cognitive impairment, developmental delay, and psychiatric diseases, such as autism spectrum disorder, besides epilepsy [138] (Figure 2). Heterozygous de novo GRIA2 (encodes Glu A2 subunit) mutations in 28 patients were reported [139]. Their phenotypes are intellectual disability, neurodevelopmental abnormalities including autism spectrum disorder, Rett syndrome-like features, and seizures or developmental epileptic encephalopathy. As previously mentioned, the GluA2 subunit is the key site for regulating calcium permeability and is affected by RNA editing as well as the structural components. In GluA2 mutant mice that lack GluA2, LTP in the CA1 region of hippocampal slices was enhanced with normal neuronal excitability [140]. The increase in Ca^2+^ permeability is a possible mechanism for enhanced LTP. The mutant mice also showed disorders in mental development and motor coordination. These results suggest an important role for GluA2 in synaptic plasticity and behavior.

### 3.3. Anti-NMDA Antibody Encephalitis

Anti-NMDA receptor encephalitis has drawn much attention over the years and is a common type of autoimmune encephalitis [141,142]. The antibody causes internalization of the receptors with a selective and reversible decrease in NMDAR surface density and synaptic localization [142]. Although patients’ antibodies reduced synaptic NMDAR-mediated currents, the localization or expression of other glutamate receptors or synaptic number were not affected [143]. It can be postulated that NMDA antagonists will reduce excitatory transmission in the brain and alleviate seizures. However, a therapeutic trial revealed that D-CPP-ene, a competitive NMDA antagonist, did not help patients with intractable complex partial seizures. Instead, it worsened seizures in three of the eight patients [144]. In the presence of patients’ cerebrospinal fluid and purified IgG antibodies, extracellular glutamate levels increased due to dysfunction of the NMDA-related glutamatergic turnover or impaired turnover of receptors, and thereby NMDA-related excitotoxicity occurred [145]. In another study, patients’ CSF and purified IgG were injected into mice brains, leading to increased extracellular glutamate [146]. This hyperglutamatergic state is speculated to be due to an imbalance between the NMDA and AMPA pathways [147]. In other words, the brain circuitry shifts to an AMPA-dependent hyperglutamatergic state under the influence of antibodies on NMDARs. It is possible that NMDAR antibody-induced elevated glutamate in the human brain is reversible and does not reach the synaptic threshold for neurodegeneration through the overactivation of AMPARs [148]. It is also accordant with the relatively better prognosis of patients with anti-NMDA encephalitis in the presence of early immunotherapy and removal of causative tumors [141,149].

To summarize, both NMDA and AMPA receptors have been associated with epilepsy and SE. Mutations or variants of subunits in NMDA and AMPA channels have been linked to various clinical features, including growth retardation, early onset of epilepsy, and psychiatric disorders. The overexpression and alteration of NMDA receptors, along with the internalization of GABA receptors, are currently considered to be potential mechanisms underlying SE, based on the evidence of the anti-seizure effects of various NMDA receptor antagonists in animal studies. Additionally, antibodies in anti-NMDA receptor encephalitis have been found to decrease the density of NMDA receptors via internalization and to increase extracellular glutamate levels, leading to excitotoxicity.

## 4. Antiseizure Medications Acting on Glutamate Receptors

It is clear that targeting the balance of excitatory and inhibitory neurotransmission is the cornerstone of antiseizure medications (ASM). Today, several ASMs acting on the glutamatergic system are available. Topiramate (TPM) has shown a broad spectrum of efficacy in drug-resistant partial and secondary generalized seizures [150,151]. In addition to blockade of voltage-dependent sodium channels and potentiation of GABA-mediated neurotransmission, it exerts antagonism in glutamate receptors. TPM inhibits neuronal excitatory pathways by selective action at AMPA and kainate receptors. TPM blocks kainate-evoked inward currents in cultured hippocampal neurons and exerts a biphasic effect on kainate-evoked currents, all of which may contribute to its anticonvulsant activity [152,153]. TPM also has a negative modulatory effect on L-type calcium channels, thus reducing the amplitude of high voltage-activated calcium currents [154].

Felbamate has a dual mechanism of action as a positive modulator of GABA_A_ receptors and an inhibitor of glutamate currents mediated by NMDARs [155]. Actually, felbamate’s anticonvulsant effect is partly mediated by antagonism at the glycine B site of NMDARs [156]. Pregabalin selectively binds to the α2δ subunit of presynaptic voltage-gated calcium channels to block P/Q-type calcium currents, and indirectly inhibits the calcium-dependent release of glutamate from vesicles [157]. Lamotrigine is categorized as a sodium channel blocker by its action on voltage-gated sodium channels. It can also modulate P/Q-type, N-type, and R-type calcium channels on presynaptic terminals [158,159], thereby inhibiting the release of glutamate.

Riluzole modulates glutamate function by inhibition of voltage-gated sodium currents to decrease presynaptic glutamate release [160], as well as by blocking Ca^2+^ entry via the NMDA channel, thereby reducing neuronal excitability [161]. Riluzole also enhances the activities of astrocytic glutamate transporters glutamate-aspartate transporter (GLAST) and glutamate transporter-1 (GLT-1), which cause buffering effects on excessive extracellular glutamate [162]. In a pilocarpine-induced or electroconvulsive shock-induced seizure model, riluzole showed its effect on seizure suppression [163,164]. Although riluzole’s efficacy has not been proven in epilepsy patients, a laminar cortex model, examining neuronal excitability and network alteration, has predicted its potential therapeutic targets for treating seizures caused by loss-of-function changes in K_v_1 channelopathy [165].

Memantine blocks ionotropic NMDA receptors by binding to Mg^2+^ binding sites when the receptor channel is open. This action inhibits the prolonged influx of Ca^2^ to stabilize NMDA activity [166]. In a pentylenetetrazole (PTZ)-induced seizure model, memantine prevented convulsions and morphological changes in rat brain neurons [167]. A mice model showed that memantine reduced cortical excitability by producing a more uniform direct current stimulation (DCS)-induced long-term depression-like activities throughout the cortical thickness. Additionally, a combination of memantine and DCS suppressed KA-induced seizures [168]. The application of memantine needs further investigations in patients with epilepsy.

As NMDA hypofunction is thought to be associated with schizophrenia, the potential adverse behavioral effects of NMDAR-selective antagonists in epilepsy treatment raise some concerns [169]. Ketamine administration in healthy men resulting in dose-dependent negative psychiatric symptoms, as well as a decrease in verbal and nonverbal declarative memory performance is an example [170]. In contrast, AMPAR signaling exerts fewer effects on neuroplasticity than NMDARs, and has greater potential to modulate hyperexcitability with incidence of psychosis [171,172]. It is for this reason that targeting modulation of AMPARs may be more promising than targeting NMDARs [173].

NMDAR antagonists provide anticonvulsant effects in the maximal electroshock and reflex seizure models [174]. They also exhibit seizure protection effects in PTZ-kindling-induced hippocampal astrocytosis, oxidative stress, and neuronal loss [175]. However, some NMDAR antagonists showed only weak anticonvulsant effects and did not increase the focal seizure threshold in fully kindled rats [176]. Instead, they may produce prominent behavioral side effects in kindled animals [177]. AMPAR antagonists are different from NMDAR antagonists in anticonvulsant activity. They have better anticonvulsant effects in fully kindled seizures, particularly having a synergistic effect with low-dose NMDA antagonists [178,179]. Furthermore, they have less behavioral side effects compared to those seen with NMDA antagonists in kindled animals. The pharmacological features of AMPAR antagonists in kindling rats suggest their potential in the treatment of partial seizures. In slices of lateral amygdala from humans with intractable TLE, blockade of AMPARs, but not NMDARs, abolished spontaneous interictal-like activity, indicating the role of AMPARs in the abnormal electrical network activity of the epileptic brain [180]. As AMPARs are involved in fast excitatory transmission, prolonged activation of these receptors is a critical process in the development and progression of epileptic seizures. Several animal studies have shown that AMPAR receptor antagonists can terminate self-sustained synchronized activity and neuroprotection in SE [181,182,183,184,185,186]. Perampanel is a noncompetitive AMPAR antagonist with a negative allosteric modulation effect [187] that decreases neuronal-synchronized epileptiform activity [172]. Perampanel has been shown to shorten the after-discharge duration in increased intensity of stimulation in the amygdala kindling model, whereas sodium channel blockers and an SV-2A ligand (levetiracetam) failed to show this effect [188]. Our previous study also expanded on perampanel’s AMPAR ionic mechanism in modulating neuronal excitability [189]. Moreover, in a recent study, perampanel, but not the NMDA antagonist amantadine, retarded development of spontaneous recurrent seizures in pilocarpine-induced SE and also reduced SE-induced late behavioral consequences [185]. Perampanel has been approved for the treatment of partial-onset epilepsy and generalized tonic-clonic epilepsy [190,191,192].

In conclusion, numerous ASMs have been found to have anti-seizure effects through various mechanisms, such as inhibiting presynaptic glutamate release and antagonizing ionotropic glutamate receptors. These medications include classic ASMs such as topiramate, felbamate, pregabalin, and lamotrigine, as well as NMDAR antagonists such as riluzole, memantine, ketamine, and AMPAR antagonists such as perampanel.

## 5. Treatment of SE by Glutamate Receptor Antagonists

Although NMDARs are targets for antiepileptic drugs, competitive NMDAR antagonists, such as D-2-amino-5-phosphonopen-tanoate (AP5) and 3-(2-carboxypiperazin-4-yl)propyl-l-phosphonate (CPP), have poor brain penetration [193,194], and their function may be dampened by the high concentrations of synaptic and extrasynaptic glutamate during epileptic seizures. Noncompetitive antagonists, such as dizocilpine (MK-801) and ketamine, have no such limitations [195]. An animal study revealed noncompetitive antagonist MK-801 to be superior to the competitive antagonist CPP and PH-sensitive site antagonist ifenprodil in terminating experimental SE [196]. Receptor trafficking is a major cause of refractory or super-refractory status epilepticus (SRSE) if treatment of SE is delayed. Intrasynaptic membrane GABA_A_ receptors are internalized, while NMDARs accumulate and are upregulated in the post-synaptic membrane, in cases of prolonged SE [113,197]. Therefore, GABAergic medications sometimes fail to control SE. In such situations, ketamine, a noncompetitive NMDA antagonist, is an option for late, refractory SE. Ketamine has been shown to play a role in the treatment of SRSE in adults and children [198,199,200,201,202,203,204]. The seizure control rate ranges from 50% to as high as 90%. In a larger retrospective series in 68 patients with SRSE under midazolam infusion, 55 patients showed more than a 50% decrease in seizure burden and 43 patients completely ceased seizures within 24 h of starting add-on ketamine treatment [205]. Their treatment regimen did not alter intracranial pressure or cerebral blood flow. In addition, the advantage of ketamine is its lack of adverse cardiopulmonary depression effect [206,207].

Magnesium sulfate is a potential therapy for SE. As mentioned earlier, Mg^2+^ blocks NMDAR at a resting state, and it is only when Mg^2+^ is unblocked that the membrane can be depolarized, allowing a voltage-dependent inward flow of sodium (Na^+^) and Ca^2+^ ions that generate excitability. Magnesium sulfate administration in rats was associated with an increased seizure threshold and resistance to experimental seizures [208]. Intravenous magnesium treatment reduced refractory epilepsy with recurrent SE and relieved bouts of epilepsia partialis continua in adolescents with POLG-1 mutation [209]. An open-label, randomized, controlled study showed significantly lengthened durations of seizure-free periods by adding magnesium sulphate to adrenocorticotropic hormone in patients with infantile spasm [210]. In pregnant women, intravenous magnesium sulphate is the standard of care in the management of eclampsia [211].

In conclusion, several candidate medications, such as competitive and noncompetitive NMDAR antagonists, have been investigated on their ability to control SE (Table 1) in animal models based on the receptor trafficking theory, and they have shown positive effects. However, the genuine efficacy of these medications in clinical studies on patients with SE remains unknown. Further clinical trials using ketamine or magnesium sulfate are warranted to precisely determine their effects on SE and RSE.

## 6. Future Perspective of ASM

Kainate receptors (KARs) are among the glutamate ionotropic receptors whose mechanism in seizures is not clearly understood. This non-NMDA receptor is upregulated in astrocytes in response to SE, as increased expression of some subunits has been noted [212]. Although the significance of increased expression of the subunits is not clear, it means that selectively targeting astrocytic processes that contribute to glutamate release may hold potential for epilepsy therapy [213]. Furthermore, selurampanel (BGG492) is an AMPA/KAR antagonist and clinical trials for its use in treating partial and photosensitive epilepsy are ongoing [214]. A 12-week phase II study revealed a higher percentage of total partial seizure reduction over placebo for BGG492 150 mg given three times a day [215].

Genetic variations in patients with epilepsy must be taken into consideration in treating epilepsy. For example, genetic variations of the CYP2B6 alleles (*1 and *6) lead to different levels of ketamine plasma concentrations. Patients with *1/*1 genotypes may accelerate clearance of ketamine, yet decreased clearance was noted in patients with the *1/*6, and particularly, *6/*6 allele [216,217]. The variation of ketamine concentration may have an impact on the efficacy of treatment and possibly serious adverse effects. Regarding GRIN2A mutation-related epileptic encephalopathy, memantine was reported to have decreased the frequency and onset of seizures in a 3-year-old boy with heterozygous c.1083G  > A (p.Leu361 =) variant in GRIN2A [218]. In a de novo missense mutation (c.2434C > A; p.L812M) patient, add-on memantine reduced seizure burden and improved interictal EEG [219]. However, we have not yet known whether memantine is effective in all of these patient populations. Some factors such as genetic variant location, mechanism of gain-of-function (or loss-of-function), patient age, memantine dose, different serum and brain concentrations might influence its efficacy [220].

Overall, there are several aspects worthy of future ASM research, including clinical trials of AMPA/KAR antagonists, the use of memantine in patients with epilepsy with specific genetic variations or mutations, and the investigation of the underlying mechanisms of how these mutations impact glutamate receptors.

## 7. Conclusions

Knowing the pathophysiology of glutamate and glutamate receptors and their interactions is essential in facilitating the clinical management of epilepsy. Different conformations of NMDARs and AMPARs, as well as different ligand substrates, have distinct types and strengths of neurotransmission. Astrocytes play an important role in regulating glutamate, either through reuptake of synaptic glutamate or release of glutamate triggered by increased intracellular Ca^2+^ levels in astrocytes. In addition, receptor trafficking in prolonged seizures leads to altered receptor expression and localization, which results in drug-resistant SE. Modulation of glutamatergic signaling by various glutamate receptor antagonists has been shown to be effective for treatment of epilepsy. New mechanisms of ASM such as targeting kainate receptors and astrocytic processes are promising. Genome sequence exploration in some epileptic encephalopathies, and evidence of genetic epilepsy could help in the development of promising personalized treatment in precision medicine.

## Figures and Tables

**Table 1:** Treatment of SE by various glutamate receptor (NMDAR) antagonists.

Medication	Mechanism	Efficacy	Remarks
Ketamine	Noncompetitive NMDA antagonism	-Treatment of SRSE in adults and children, with control rate ranging from 50% to as high as 90%	-Does not alter intracranial pressure or cerebral blood flow -Lack of adverse cardiopulmonary depression effect
Magnesium sulfate	NMDA antagonism	-Associated with an increased seizure threshold and reduced seizures in animal studies; effective add-on therapy in infantile spasm	-Intravenous magnesium sulphate is the standard management in eclamptic seizures.
Dizocilpine (MK-801)	Noncompetitive NMDA antagonism	-In SE animal models, MK-801 is superior to the competitive antagonist CPP and noncompetitive antagonist ifenprodil	

## Data Availability

Not applicable.
